# A Broad-Based Mosquito Yeast Interfering RNA Pesticide Targeting *Rbfox1* Represses *Notch* Signaling and Kills Both Larvae and Adult Mosquitoes

**DOI:** 10.3390/pathogens10101251

**Published:** 2021-09-28

**Authors:** Keshava Mysore, Longhua Sun, Limb K. Hapairai, Chien-Wei Wang, Joseph B. Roethele, Jessica Igiede, Max P. Scheel, Nicholas D. Scheel, Ping Li, Na Wei, David W. Severson, Molly Duman-Scheel

**Affiliations:** 1 Department of Medical and Molecular Genetics, Indiana University School of Medicine, Raclin-Carmichael Hall, 1234 Notre Dame Ave., South Bend, IN 46617, USA; kmysore@iu.edu (K.M.); Longhua.Sun.15@nd.edu (L.S.); limbh@pihoa.org (L.K.H.); jroethe@iu.edu (J.B.R.); mpscheel@iu.edu (M.P.S.); pli2@nd.edu (P.L.); Severson.1@nd.edu (D.W.S.); 2 Eck Institute for Global Health, The University of Notre Dame, South Bend, IN 46556, USA; cwang16@nd.edu (C.-W.W.); jigiede@nd.edu (J.I.); nscheel@iu.edu (N.D.S.); nwei@nd.edu (N.W.); 3 Department of Civil and Environmental Engineering and Earth Sciences, The University of Notre Dame, South Bend, IN 46556, USA; 4 Department of Biological Sciences, The University of Notre Dame, South Bend, IN 46556, USA; 5 Department of Life Sciences, The University of the West Indies, St. Augustine, Trinidad and Tobago

**Keywords:** *Aedes aegypti*, *Aedes albopictus*, *Culex quinquefasciatus*, *Anopheles gambiae*, *Saccharomyces cerevisiae*, RNAi, insecticide, larvicide, adulticide, yeast

## Abstract

Prevention of mosquito-borne infectious diseases will require new classes of environmentally safe insecticides and novel mosquito control technologies. *Saccharomyces cerevisiae* was engineered to express short hairpin RNA (shRNA) corresponding to mosquito *Rbfox1* genes. The yeast induced target gene silencing, resulting in larval death that was observed in both laboratory and outdoor semi-field trials conducted on *Aedes aegypti*. High levels of mortality were also observed during simulated field trials in which adult females consumed yeast delivered through a sugar bait. Mortality correlated with defects in the mosquito brain, in which a role for *Rbfox1* as a positive regulator of *Notch* signaling was identified. The larvicidal and adulticidal activities of the yeast were subsequently confirmed in trials conducted on *Aedes albopictus*, *Anopheles gambiae*, and *Culex quinquefasciatus*, yet the yeast had no impact on survival of select non-target arthropods. These studies indicate that yeast RNAi pesticides targeting *Rbfox1* could be further developed as broad-based mosquito larvicides and adulticides for deployment in integrated biorational mosquito control programs. These findings also suggest that the species-specificity of attractive targeted sugar baits, a new paradigm for vector control, could potentially be enhanced through RNAi technology, and specifically through the use of yeast-based interfering RNA pesticides.

## 1. Introduction

Although vector control is the primary mechanism for mosquito-borne disease prevention, resistance to all classes of chemical insecticides has been documented worldwide in a variety of disease vector mosquitoes [1]. The potential for unintended deleterious impacts of insecticides on non-target target organisms, including humans, is also of concern and is continuously monitored [2]. Given these issues, the successful prevention of arthropod-borne infectious diseases will require new classes of environmentally safe insecticides and novel mosquito control technologies [3]. RNAi, an innate eukaryotic regulatory pathway that silences gene expression through the generation of small interfering RNA (siRNA), has been successfully applied in the laboratory for the functional characterization of genes in many species, including mosquitoes [1,4]. Recent efforts have focused on the potential translation of RNAi technology, which has attracted interest in the agricultural pest control community [5], to the field for mosquito control [1,4]. Laboratory screens [6,7] led to the discovery of siRNAs which correspond to genes that are required for mosquito larval survival. Several of these siRNAs target genes that are required at multiple stages of the mosquito life cycle, thereby functioning as both larvicides and adulticides [8,9]. Moreover, a subgroup of these siRNAs correspond to genetic target sites that are conserved in *Aedes* (dengue, Zika, yellow fever and chikungunya vector), *Culex* (West Nile and lymphatic filariasis vector), and *Anopheles* (malaria vector) species, but which have not yet been found to be identically conserved in the sequenced genomes of non-target organisms [9,10,11]. It was hypothesized that these interfering RNA pesticides (IRPs) will kill multiple types of mosquitoes during the larval and adult stages, yet pose little or no threat to non-target organisms. To further evaluate this hypothesis, the present investigation pursued characterization of a putative adulticidal and larvicidal IRP with a conserved target site in mosquito *Rbfox1* genes.

*Rbfox1* genes, which are also known as *Ataxin 2-binding protein* (*A2BP1*) genes, encode evolutionarily conserved RNA binding proteins that function as critical regulators of neural and muscular development in divergent species [12,13,14]. Human A2BP1 was identified as an Ataxin 2-associated nuclear RNA-binding protein that was found to be linked to type 2 spinocerebellar ataxia [15] and which has been associated with a number of other human disorders, including autism, epilepsy, and cardiac hypertrophy [12]. A conserved RRM domain facilitates sequence-specific binding to UGCAUG motifs located in pre-mRNA introns, 3′ UTRS, and pre-miRNA hairpins, permitting the regulation of splicing, mRNA stability, translation, and the processing of miRNAs by Rbfox1 proteins [12,16]. Rbfox1 also promotes ribonucleoprotein granule formation and cell survival [17]. In *Drosophila melanogaster,* Rbfox1 is known to regulate germ cell development [18,19] wing development [20,21], memory [22], the cellular immune response [23], and neurogenesis [13]. In the developing nervous system of *D. melanogaster*, the role of Rbfox1 has been best characterized in the developing sensory system [13], in which Rbfox1 was found to promote sensory organ specification by potentiating *Notch* signaling. Rbfox1 was proposed to function as part of the Suppressor of Hairless complex, which in the presence of activated *Notch* (N), regulates expression of Enhancer of split-Complex (E(spl)-C) target genes [13]. Loss of *Rbfox1* expression in the early fly embryo was linked to a reduction in neuronal cell number [24], suggesting that Rbfox1 regulates *Notch* signaling at multiple stages of *D. melanogaster* development.

In this investigation, the effects of silencing the *Rbfox1* gene in multiple species of disease vector mosquitoes was examined. Loss of *Rbfox1* has been associated with larval and adult death in *D. melanogaster* [21,25], supporting the hypothesis, which was examined here through RNAi-mediated silencing experiments, that loss of *Rbfox1* function in mosquitoes could result in death at multiple stages of the mosquito life cycle. This study also describes, for the first time, the development of a yeast IRP-based ATSB system for control of adult mosquitoes. The results of these studies suggest that IRPs directed against *Rbfox1* genes may represent a new biorational intervention that could one day be applied for control of multiple species of disease vector mosquitoes at different stages of the mosquito life cycle.

## 2. Results and Discussion

### 2.1. Silencing Rbfox1 Results in A. aegypti Larval Mortality

Rbfox1.457 siRNA corresponds to a target sequence in *Rbfox1* that is conserved in multiple species of disease vector mosquitoes (Table S1) [26]. With the exception of mosquitoes, this target site was not identified in sequenced genomes [27] other than that of the disease vector sand flies *Lutzomyia longipalpus* and *Phlebotomus papatasi* [28], or the black soldier fly *Hermetia illucens* (Table S1). Rbfox1.457 siRNA was used to examine if the *Rbfox1* gene is required for mosquito viability. The siRNA was evaluated in *A. aegypti*, in which it induced significant fourth instar larval mortality following brief soaking treatments in 0.5 µg/µL Rbfox1.457 siRNA (Table 1). Likewise, significant mortality was observed in *A. aegypti* adult females within six days following microinjection of Rbfox1.457 siRNA into the adult thorax (Table 1). The results of these studies demonstrated that this siRNA has both larvicidal and adulticidal activities in *A. aegypti*, supporting the hypothesis that *A. aegypti*, like *D. melanogaster*, requires Rbfox1 activity at multiple stages of the mosquito life cycle.

### 2.2. Sugar Baited Delivery of Rbfox1.457 siRNA 

The identification of field-appropriate mechanisms for delivery of IRPs to mosquitoes has been central to ongoing research efforts [4]. Attractive targeted sugar baits (ATSBs) are a new paradigm for mosquito control that exploits the innate sugar feeding behavior of female and male mosquitoes, which typically feed on natural sugar sources [29]. This sugar feeding behavior permits mosquito control through deployment of sugar baits that have been laced with insecticide. Although sugar baits can facilitate targeted delivery of a variety of insecticides, insecticide resistance is nevertheless of concern [30], and the development of new classes of pesticides, such as IRPs, which could one day be delivered as second-generation ATSBs, may therefore be advantageous. To this end, Rbfox1.457 siRNA was evaluated in a simulated field study using a sugar bait delivery system which had been described previously [8,9]. Adult female mosquitoes were permitted to feed on sugar bait alone, sugar bait with control siRNA, or sugar bait with Rbfox1.457 siRNA. Feeding rates among *A. aegypti* females, which are shown in Table 2, were comparable to those reported in similar trials with other siRNAs [8,9]. As previously observed, no significant differences in *A. aegypti* feeding rates were detected among the various treatments (*p* > 0.05). Although adult mortality among mosquitoes fed with sugar bait alone or control siRNA sugar bait was negligible (Table 1), Rbfox1.457 siRNA ATSB feedings resulted in significant mortality in *A. aegypti* adult females, with 77 ± 7.1% mortality detected within six days following consumption of Rbfox1.457 siRNA sugar meals (Table 1).

### 2.3. Development of an RNAi-Based Yeast Pesticide That Can Be Deployed as an ATSB

**Figure 1:**
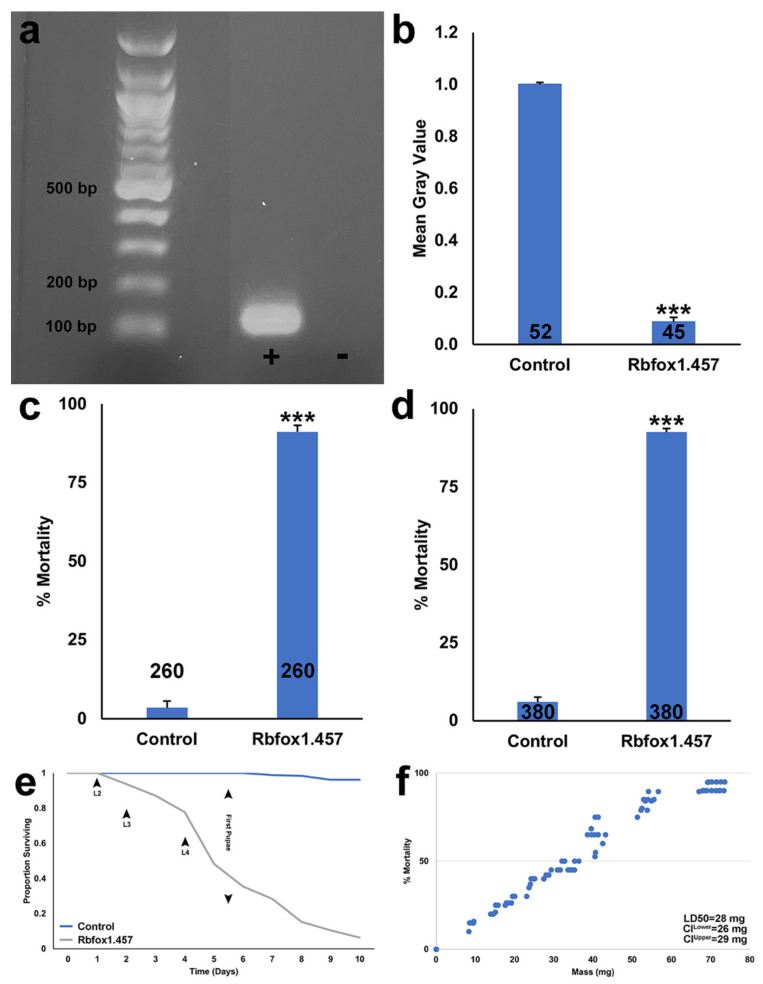
Rbfox1.457 yeast consumption results in *Rbfox1* silencing and *A. aegypti* larval death. (**a**) A ~100 bp PCR amplicon generated using primers corresponding to the Rbfox1.457 shRNA transcript is visualized in the lane marked by + in an ethidium bromide-stained agarose gel. cDNA template for the reaction was prepared from Rbfox1.457 yeast total RNA, and a negative control PCR reaction with no cDNA added (marked by a minus symbol) is included at far right. A representative gel from two comparable biological replicate experiments is shown; irrelevant lanes between a DNA standard at far left and the + lane were cropped from an image of the original gel, which is included as supplementary material. (**b**) *Rbfox1* transcripts detected in the *A. aegypti* L4 brain were significantly reduced in larvae fed with dried inactivated yeast interfering RNA larvicide Rbfox1.457 tablets. Compiled mean gray value results provide evidence of RNAi-mediated silencing; n numbers are indicated below each bar in the graphs. (**c**) *A. aegypti* larval consumption of inactivated dried Rbfox1.457 yeast resulted in significant larval death in laboratory trials. (**d**) Rbfox1.457 induced significant larval lethality in outdoor semi-field trials conducted in 7.5 L containers bearing 3.5 L water. In (**c**,**d**), data compiled from multiple replicate trials (each with 20 larvae) are represented as mean percentages of larval mortality; error bars denote SEM, and n numbers are indicated below each bar in the graphs. (**e**) A survival-curve for *A. aegypti* larvae reared on the indicated diets is shown, indicating that a majority of Rbfox1.457 yeast-treated larvae died by the fourth instar of larval development. (**f**) A dose response curve illustrates that larval mortality is correlated to the amount of Rbfox1.457 yeast consumed. Each data point corresponds to the percent mortality observed in a single-container assay conducted with 20 larvae. *** = *p* < 0.001 (Student’s *t*-test).

Although the high rates of mortality induced by Rbfox1.457 siRNAs suggest that this technology could facilitate mosquito control, the high cost of siRNAs could impede broad use of siRNA pesticides in the field [4]. To address this, *S. cerevisiae* has been used to express shRNAs corresponding to insecticidal siRNAs, facilitating cost-efficient interfering RNA production during yeast cultivation [31]. Genetically modified strains in which shRNA has been expressed can be cultured, then heat-inactivated, dried, and fed to mosquito larvae [6], suggesting that it might also be possible to engineer Rbfox1.457 yeast that could be used in the development of yeast IRP-based ATSBs for adult mosquito control. To this end, stably transformed *S. cerevisiae* expressing shRNA corresponding to Rbfox1.457 siRNA (hereafter referred to as Rbfox1.457 yeast) was generated by integrating two Rbfox1.457 shRNA expression cassettes into *S. cerevisiae.* shRNA expression was confirmed through amplification of cDNA generated from total RNA that had been prepared from the yeast strain (Figure 1a).

Prior to using the Rbfox1.457 strain in the development of a yeast ATSB system, the insecticidal activity of this strain was first confirmed in larvae. Larval consumption of this yeast led to a 91 ± 2% reduction in *Rbfox1* transcripts in the *A. aegypti* larval brain (Figure 1b). Consumption of the yeast also resulted in 91 ± 2% larval mortality with respect to larvae reared on control interfering RNA yeast, in which shRNA with no known target in mosquitoes had been expressed (Figure 1c; *p* < 0.001 vs. control). A majority of treated larvae died within eight days by the fourth instar of larval development, while larvae consuming the control yeast survived until adulthood (see survival curve in Figure 1e). The dosage of Rbfox1.457 correlated directly with the percentage of larval mortality (Figure 1f), with the LD_50_ determined to be 28 mg. Larvicide activity was also verified in semi-field trials conducted at an outdoor rooftop laboratory in Notre Dame, IN, where 93 ± 1% larval mortality was observed following Rbfox1.457 treatment (Figure 1d; *p* < 0.001 vs. control). These studies, combined with previous studies in which yeast IRP larvicides were shown to function in different types of water [10,11] and in an assortment of different sized containers with varied water volumes and larval densities [6,10,11,32,33] provide further evidence that yeast IRPs may represent a new larvicidal intervention, adding Rbfox1.457 to the growing arsenal of yeast larvicides.

**Figure 2:**
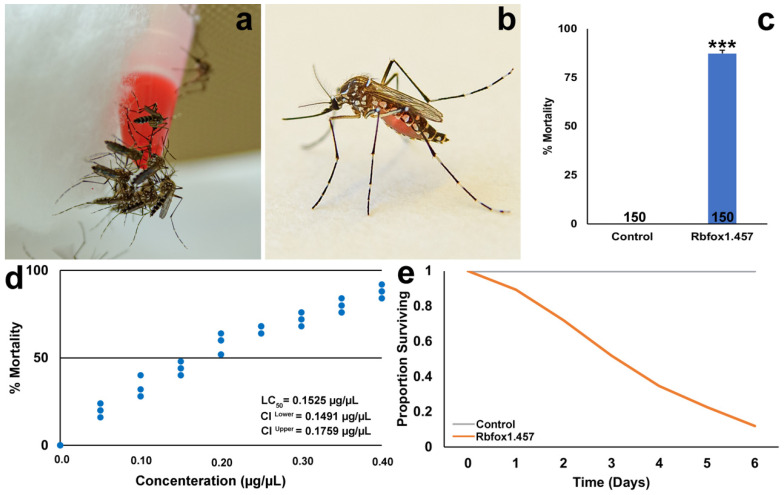
RNAi-based ATSBs targeting *Rbfox1* result in high levels of *A. aegypti* mortality. (**a**) Heat-inactivated Rbfox1.457 yeast can be delivered in 5% sucrose as an ATSB (red-dyed solution at feeding station in (**a**) that is readily consumed by *A. aegypti* adult females. (**b**) An engorged female that has consumed red ATSB is shown. (**c**) Significant mortality is observed in *A. aegypti* that consumed Rbfox1.457 yeast ATSB in comparison to adults that consumed sugar bait with control yeast. *** = *p* < 0.001; error bars represent SEM; n numbers are indicated below each bar in the graph. (**d**) A dose-response curve showing the concentration of Rbfox1.457 yeast in the sugar bait vs. the percentage mortality of *A. aegypti* adult females is shown; each point represents an ATSB trial with 25 adult females. (**e**) The survival curve for adult females that fed on control yeast sugar bait or Rbfox1.457 yeast sugar bait is shown.

Based on these findings in larvae, Rbfox1.457 yeast was used in the development of an Rbfox1.457 ATSB-based yeast delivery system for targeting adult mosquitoes. Inactivated, lyophilized, powdered yeast was added to red-dyed sugar-bait solutions. Gellan gum was included to help prevent yeast ATSB desiccation, and benzoic acid was used as a preservative to hinder growth of contaminating microbes in the ATSB, which was delivered in small centrifuge tubes that had been cut, exposing a droplet of yeast ATSB from the tubes. The resulting yeast ATSB feeders were hung in mosquito cages, in which *A. aegypti* adult females congregated to and drank from the feeders (Figure 2a). Highly increased feeding rates were observed with respect to 4-h delivery of siRNA-ATSBs (Table 2), with yeast ATSB consumption (which was tracked through the presence of red dye in the abdomens of engorged females, Figure 2b) confirmed in 100% of adult female mosquitoes (Table 2). A mortality of 87 ± 2% was observed in mosquitoes that consumed Rbfox1.457 yeast in 5% sucrose sugar bait solution (Figure 2c, *p* < 0.001 with respect to control yeast in 5% sugar bait). Rbfox1.457 yeast killed a majority of *A. aegypti* mosquitoes within 3–4 days of initiating treatment (Figure 2e). The percentage mortality correlated with the concentration of Rbfox1.457 yeast in the ATSB (Figure 2d; LC_50_ = 0.1525 µg/µL ATSB). It is possible that generation of yeast strains that express higher levels of shRNA could lead to even higher mortality rates, and this possibility can be explored in future studies. However, it will be interesting to repeat these studies in the field, where mortality rates could increase to 100% when mosquitoes are subjected to IRP treatments in conjunction with environmental stresses. 

### 2.4. Silencing Rbfox1 Represses Notch Signaling

**Figure 3:**
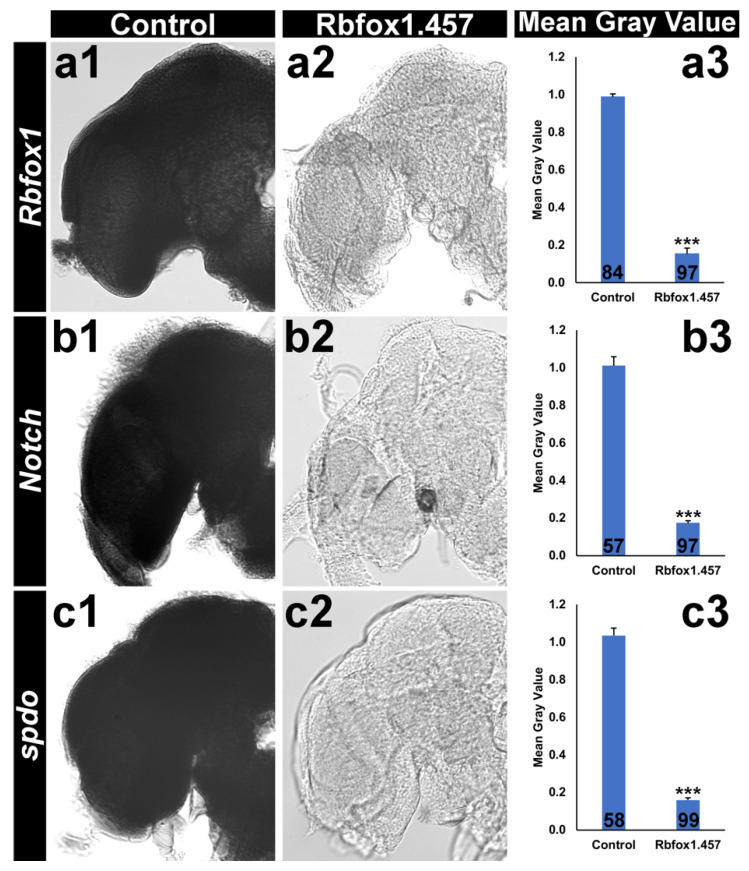
Rbfox1.457 yeast ATSB results in target gene silencing and significantly impacts *Notch* signaling in *A. aegypti.* Rbfox1.457 ATSB consumption resulted in a significant reduction in *Rbfox1* (**a**), *Notch* (**b**), and *spdo* (**c**) transcripts in the *A. aegypti* adult female brain; n numbers of brains analyzed are indicated below each bar in the graphs. *** = *p* < 0.001 vs. sugar bait alone or sugar bait with control yeast; data were analyzed with Student’s *t*-test.

The mode of action for Rbfox1 IRPs was next examined. As noted above, Rbfox1 is known to be required in a variety of different tissues, including the nervous system [13]. Given that many of the lethal IRPs characterized in recent years have significant impacts on the mosquito central nervous system (CNS; [6,7,8]), this study focused on characterizing the role of Rbfox1 in the mosquito brain. Rbfox1 potentiates *Notch* signaling in the *D. melanogaster* sensory system [13], suggesting that Rbfox1 could function as a potentiator of *Notch* signaling in the mosquito brain. Silencing *Rbfox1* transcripts in the *A. aegypti* female brain (Figure 3(a1–a3), *p* < 0.001 vs. control interfering RNA yeast-treated females) led to a significant decrease in *Notch* transcripts (Figure 3(b1–b3)), *p* < 0.001 vs. control interfering RNA yeast-fed females). Expression of *sanpodo* (*spdo*), another key component of the *Notch* signaling pathway [34], was also assessed. A significant 84 ± 1% reduction in *spdo* transcripts was detected in adult females following *Rbfox1* silencing (Figure 3(c1–c3), *p* < 0.001). This significant loss of *Notch* and *spdo* expression suggest that loss of viability following *Rbfox1* silencing results, at least in part, from loss of critical *Notch* signaling in the mosquito CNS. These results suggest that in addition to regulating E(spl)-C target genes [13], Rbfox1 impacts the expression of *Notch* itself, as well as *spdo*. These impacts on *Notch* and *spdo* expression, which have not to our knowledge been reported in the past, could have potential implications for the known associations of Rbfox1/A2BP1 to human disease states [12], a topic that could be further explored.

### 2.5. Rbfox1.457 Yeast Pesticides Function as Broad-Range Mosquito Insecticides but Are Not Found to Be Toxic to Select Non-Target Arthropods

**Figure 4:**
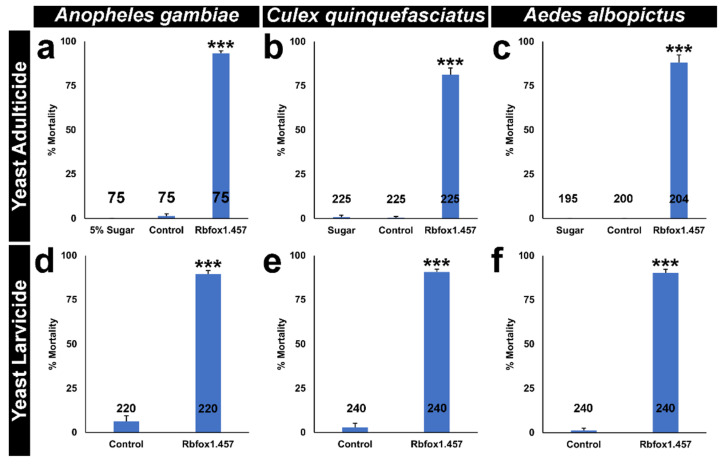
Rbfox1.457 yeast is a broad-based mosquito adulticide and larvicide. Oral consumption of Rbfox1.457 yeast ATSB results in high levels of adult (**a**–**c**) and larval (**d**–**f**) mortality in *A. gambiae* (**a**,**d**), *C. quinquefasciatus* (**b**,**e**), and *A. albopictus* (**c**,**f**). The data represent mean mortalities with error bars representing SEM; n numbers are indicated below each bar in the graphs. Data were statistically analyzed with ANOVA in (**a**–**c**) and Student’s *t*-test in (**d**–**f**). *** *p* < 0.001 in comparison to control-treated individuals.

The target site of Rbfox1 IRPs is identically conserved in multiple species of disease vector mosquitoes, including multiple *Anopheles* spp., *A. albopictus*, and *C. quinquefasciatus* (Table S1). Based on the results obtained for *A. aegypti* (Figure 1 and Figure 2), it was therefore hypothesized that Rbfox1.457 yeast could function as a broad-range mosquito IRP that can kill both adults and larvae of multiple mosquito species. To evaluate this hypothesis, Rbfox1.457 yeast ATSB was assessed in *A. gambiae,* in which 100% feeding rates, which were comparable to those observed for Rbfox1.457 yeast in *A. aegypti,* were noted (Table 2). 93 ± 1% adult mortality was observed in *A. gambiae* adult females that had consumed Rbfox1.457 ATSB (Figure 4a, *p* < 0.001 compared to control-yeast treated adults in which only 1 ± 1% mortality was observed). As observed in *A. aegypti,* silencing of *Rbfox1* in *A. gambiae* (Figure S1(a1–a3)) resulted in decreased expression of *A. gambiae Notch* (Figure S1(b1–b3)) and *spdo* transcripts (Figure S1(c1–c3)). Rbfox1.457 yeast consumption also resulted in significant 89 ± 4% mortality in *A. albopictus* (Figure 4c, *p* < 0.001 vs. control-yeast treated adults) and 81 ± 4% *C. quinquefasciatus* (Figure 4b, *p* < 0.001 vs. control-yeast treated adults), with high female feeding rates (90% and 100%, respectively) observed in these species (Table 2). 

Likewise, Rbfox1.457 yeast treatments resulted in 90 ± 2% larval mortality in *A. gambiae* larvae (Figure 4d, *p* < 0.001 vs. control yeast treatment), 91 ± 2% mortality in *C. quinquefasciatus* larvae (Figure 4e; *p* < 0.001 vs. control yeast treatment), and 90 ± 2% larval mortality in *A. albopictus* (Figure 4f, *p* < 0.001 vs. control yeast treatment). As observed with other yeast IRPs [10,11], 100% mortality was achieved in some, but not all containers. Although this raises the question of whether a proportion of the mosquitoes are resistant to RNAi, no loss of sensitivity to yeast IRPs has been observed in studies to date. Instead, previous work indicated that 100% killing rates are induced by yeast IRPs when larvae are reared as individuals, preventing consumption of dead larvae in the container and resulting in higher kill rates [10,11], a phenomenon that should be investigated in future field trials.

Although Rbfox1.457 IRPs kill larvae and adults of several mosquito species (Figure 1, Figure 2 and Figure 4), these IRPs were not found to have activity in several non-target arthropods, including the crustacean *Daphnia magna,* the fruit fly *D. melanogaster, Hippodamia convergens* (lady beetle)*, Oncopeltus fasciatus* (milkweed bug), or *Tribolium castaneum* (flour beetle; Table 3). Cumulatively, these data support the hypothesis that Rbfox1.457 IRPs function as broad-based mosquito insecticides that kill both adults and larvae, yet have activity that poses little, if any threat to non-target species. If Rbfox1.457 IRPs are commercialized, it will be critical to pursue further toxicity testing in fish and other vertebrates in support of registry applications to the EPA or other regulatory bodies. Such testing can be pursued when commercial-ready insecticide formulations have been further developed and optimized. 

Larviciding is a critical component of *Aedes* and *Culex* mosquito control programs, and the potential for adding a new class of larvicides to these programs is of interest, particularly given concerns for the emergence of resistance to existing larvicide classes [3,35,36,37]. Although *Anopheles* mosquito control programs have largely centered on targeting adults, larviciding is recommended in instances in which *Anopheles* breeding sites are fixed, few, and findable [36]. In recent years, long-lasting Bti larvicides are showing promise for control of *A. gambiae* and *A. funestus* larvae [38,39,40,41]. Moreover, the observation that *Anopheles stephensi* and *A. aegypti* share breeding containers [42] suggests that use of a larvicide that is capable of killing both *Aedes* and *Anopheles* larvae would be useful.

Adult control is also important for *Aedes* and *Culex* integrated mosquitoes control programs, for which ATSB technologies are presently being assessed [29]. ATSB-mediated delivery of a variety of broad-based adulticides, for example boric acid, encapsulated garlic oil, dinotefuran, and eugenol, have been applied for successful targeting of *A. aegypti*, *A. albopictus,* and other *Aedes* species [43,44,45,46,47,48,49]. Likewise, *C. quinquefasciatus, Culex pipiens, Culex tarsalis,* and a variety of other *Culex* species, including nuisance biters, have been successfully targeted with ATSBs containing insecticides such as boric acid, dinotefuran, eugenol, Spinosad, and encapsulated garlic oil [44,50,51,52]. ATSBs are also demonstrating great promise for targeting *Anopheles* malaria vector mosquitoes [53,54,55,56,57]. Despite these overwhelmingly positive findings, challenges for adoption and long-term use of this technology remain. Although ATSBs facilitate targeted delivery of a variety of pesticides, insecticide resistance is still a concern [29]. Furthermore, while the addition of protective membrane barriers to bait stations [58], as well as efforts to limit ATSB applications to non-flowering vegetation have decreased the potential for harming non-target organisms, it is difficult to completely eliminate all non-target risks associated with most ATSB formulations, which are not specific to mosquitoes [29]. Mosquito-specific biorational yeast IRPs could therefore hold the potential to significantly enhance ATSB technology. IRPs have a highly desirable safety profile, particularly when compared to many currently used conventional pesticides [59], and the lack of Rbfox1.457 toxicity observed in select non-target arthropods (Table 3) suggests that this yeast IRP could enhance the species-specificity of ATSBs. 

As noted above, outside of mosquitoes, the Rbfox1.457 target sequence was identified in the sand flies *Lutzomyia longipalpus* and *Phlebotomus papatasi* (Table S1), which vector parasites that cause visceral leishmaniasis [28]. Although Rbfox1.457 IRPs could potentially kill sand fly larvae, the breeding sites of sand flies are diverse [60,61], and it may therefore be more straightforward to use the yeast to target adults. Sugar baits that impact sand fly viability and which block parasite transmission are being developed and evaluated in *L. longipalpus* with the goal of conceptualizing new strategies for controlling this vector [62,63]. It would therefore be interesting to evaluate Rbfox1.457 yeast sugar baits in sand flies. Based on results observed in mosquitoes, the yeast adulticidal activity may be faster-acting than beta-glycosidic ATSBs that were previously evaluated [62], yet potentially retain the environmentally-friendly nature of these insecticides. It would also be interesting to evaluate if the increased consumption of yeast-based ATSBs observed in mosquitoes is retained in sand flies. 

## 3. Materials and Methods

### 3.1. Mosquito Rearing

Mosquito strains used in this study included: *A. albopictus* Gainesville (BEI Resources, NIAID, NIH: MRA-804, contributed by Sandra A. Allan), *A*. *aegypti* Liverpool-IB12 (LVP-IB12), *A. gambiae* G3 (BEI Resources, NIAID, NIH: Eggs, MRA-112, contributed by Mark Q. Benedict), and *C. quinquefasciatus* JHB (provided by the Centers for Disease Control and Prevention for distribution by BEI Resources, NIAID, NIH: Eggs, NR-43025). These mosquitoes were reared as described [64] in the insectary, which was maintained at 26.5 °C, with a 12 h dark/12 h light cycle that included 1 h crepuscular periods at the beginning and end of each cycle, and at ~80% relative humidity. An artificial membrane (Hemotek Limited, Blackburn, UK) was used to deliver commercially acquired sheep blood (HemoStat Laboratories, Dixon, CA, USA). 

### 3.2. Identification of siRNA #457 

siRNA #457, which corresponds to a conserved target sequence identified in the *Rbfox1* genes of many disease vector mosquitoes (see gene identification numbers in Table S1) was assessed in *A. aegypti* through larval soaking [6,7] and adult microinjection studies [8,9] as described. In summary, larval soaking was performed per the Singh et al. [65] protocol using 20 μL of 0.5 μg/μL siRNA to treat first instar (L1) larvae for four hours. These soaking trials were performed in duplicate experiments, each which were conducted on 20 larvae (n = 40 larvae evaluated in total per treatment) that were reared to adulthood and assessed as described in the World Health Organization (WHO) larvicide testing guidelines [66] following soaking treatments. Data were evaluated using the Fisher’s exact test. siRNAs used in these studies were purchased from Integrated DNA Technologies (Coralville, IA, USA) and corresponded to the following sequences #457: 5′-UAAUAGUAGCGAUGCGGAGCGAGCA-3′ in *Rbfox1* (*AAEL019934*), as well as a control sequence that does not have a known target in mosquitoes [67]: 5′-GAAGAGCACUGAUAGAUGUUAGCGU-3′. For analysis of the adulticidal activity, as previously detailed [6,7], three-day old non-blood fed adult females were anesthetized with carbon dioxide gas and microinjected in the thoracic region with 250 nL of 9 μg/μL Rbfox1.457 or control siRNA. Adults were placed in a cage to recover following injections, and adult mortality was assessed daily for the next week. Twenty individuals were injected per treatment in each of four total replicate experiments. Microinjection data were analyzed using the Fisher’s exact test. 

### 3.3. Adult siRNA-ATSB Trials

ATSB trials with siRNA were performed as detailed previously [8,9]. In summary, baits consisted of 64 μL of 5% sucrose solution (in sterile DEPC-treated water) containing 0.5% of red tracer dye (McCormick) alone or with 2.5 μg/μL of control or Rbfox1.457 siRNA delivered from a cotton wick in a cut 0.2 mL microfuge tube placed in a 3.75 L cage (Berry Global, Evansville, IN, USA). In each of 3 trials, 25 4–5-day-old adult females that had not blood fed were sugar starved for 48 h prior to four-hour sugar bait feedings carried out at dawn. Engorged females were collected as individuals, and survival was scored daily for six days. Three biological replicate experiments were conducted (n = 75 adult females in total assessed per treatment). Feeding rates were evaluated with the G-test of independence, and the log-rank test was used to compare survival rates among treatments.

### 3.4. Generation of Yeast Interfering RNA Larvicide Strains and Yeast Culturing

Custom DNA oligonucleotides encoding an shRNA expression cassette which corresponds to the Rbfox1.457 target site, 5′-TAATAGTAGCGATGCGGAGCG-3′, were purchased from Invitrogen Life Technologies (Carlsbad, CA, USA) and used to generate transformants with the shRNA expression cassette stably integrated into the *TRP1* and *URA3* sites of the *S. cerevisiae CEN.PK* strain [genotype *MAT*a/α *ura3-52*/*ura3-52 trp1-289*/*trp1-289 leu2-3_112*/*leu2-3_112 his3* Δ*1*/*his3* Δ*1 MAL2-8C/MAL2-8C SUC2/SUC2* [68]] yeast using previously described methodology [6]. This strain, hereafter referred to as Rbfox1.457 yeast IRP, as well as a comparable control shRNA expression strain [6] that had been constructed previously, which corresponds to the control siRNA sequence, were cultured as described [6]. Dried inactivated yeast interfering RNA tablets were prepared as previously described for use in larvicide assays [69]. Yeast utilized in ATSB trials was cultured in a similar manner, except that the yeast was pelleted and subsequently lyophilized using a Labconco FreeZone 6 L Console Freeze Dryer following culturing.

### 3.5. Larvicide Trials

#### 3.5.1. Laboratory Assays

Larvicide trials that conformed to the WHO larvicide testing guidelines [66] were performed in the insectary as previously described [69]. Thirteen replicate container trials were performed, each with 20 first instar larvae (n = 260 larvae total per treatment) that had been placed in 50 mL of distilled water in a 500 mL container. In each container, larvae were provided with a single 50 mg yeast tablet (either Rbfox1.457 or control) that was provided at the onset of each trial, and which was sufficient to permit feeding ad libitum throughout the trial. Containers were assessed for larval mortality throughout the trial period, and the percentages of larval mortality were transformed using arcsine transformation as described [66] at the conclusion of each trial, with data analyzed using the Student’s *t*-test. 

Previously described methodology [6,7] was used to generate dose–response curves following analysis of a variety of yeast dosages (n = 180 larvae per dosage). Dose–response data were analyzed using SPSS 25 software (IMB, Armonk, NY, USA) and log dosage–probit mortality to generate LD_50_ values with 95% confidence intervals. 

#### 3.5.2. Semi-Field Trials

Semi-field larvicide trials were performed in accordance with the WHO larvicide testing guidelines [66] in June and July 2019 at an outdoor rooftop laboratory in Notre Dame, IN as previously described [10,11]. LVP-IB12 strain *A. aegypti* mosquitoes were used in these assays, in which larvae were placed in 7.5 L containers (diameter = 23 cm, height = 25 cm). Nineteen replicate container trials, each with 20 larvae, were assessed per yeast treatment (n = 380 larvae per treatment assessed in total). The percentages of larval mortality were transformed using arcsine transformation, and data from multiple replicate experiments were assessed with a Student’s *t*-test. Outdoor temperatures ranged from 9 °C to 35 °C during the trial period, with a mean daytime temperature of 23.5 ± 5 °C and nighttime temperature of 19 ± 4 °C and an average 75 ± 15% relative humidity level.

#### 3.5.3. Yeast ATSB Simulated Field Trials

The yeast ATSB solution was prepared by mixing 40 mg of lyophilized yeast (Rbfox1.457 or control) containing 0.1% benzoic acid with 100 μL of a gellan gum (Phytagel brand from Sigma Aldrich, St. Louis, MO, USA) and sucrose stock solution containing 4.5 μL of red tracer dye (McCormick’s) in a 1.5 mL microfuge tube; for *A. gambiae* experiments, the amount of yeast was reduced from 40 to 20 mg per 100 μL of ATSB, as adults of this species are smaller in size and require less interfering RNA [8,9]. The gellan gum and sugar stock solution used in this mixture was prepared by heating 60 mL of autoclaved distilled water on a hot plate to 65 °C with constant stirring, to which 0.05 g of gellan gum, was added and the temperature increased to 90 °C while maintaining stirring. After the gellan gum was in solution (~20 min), 5 g of sucrose was added to the solution, which was then cooled to 60 °C, and the total volume brought to 100 mL through the addition of water. To prepare the feeders, the bottom of the microfuge tube was scored with a razor blade without puncturing the tube, which was subsequently capped and punctured using a thumbtack before hanging the yeast wick feeder facing down at the top of the experimental cage. Twenty-five non-blood fed 5–6-day-old adult females that had been sugar starved for 48 h were allowed to feed for four hours from two feeders placed in 3.75 L insect cages. Following confirmation of engorgement, survival was scored daily for six days. These trials were performed in triplicate. Feeding rates were evaluated with the G-test of independence, and ANOVA was used to compare survival rates. Dose-response curves for Rbfox1.457 yeast ATSB were produced and evaluated as described above for the yeast larvicides.

### 3.6. Whole Mount In Situ Hybridization 

The Patel [70] protocol was used for synthesis of riboprobes corresponding to the *A. aegypti Rbf1* (*AAEL019934*), *Notch* (*AAEL023745*), and *spdo* (*AAEL026911*) genes, as well as the *A. gambiae Rbf1* (Table S1), *Notch* (*AGAP001015*), and *spdo* (*AGAP002689*) genes and used for in situ hybridization experiments that were performed on adult female brains as previously described [71]. Four biological replicate experiments were conducted on larvae that had been fed with control or Rbfox1.457 yeasts as described above. Following processing, the brain tissues were mounted, then viewed and imaged with a Zeiss Axioimager (Carl Zeiss Microscopy, LLC, Thornwood, NY, USA) equipped with a Spot Flex camera (Diagnostic Instruments, Inc. Sterling Heights, MI, USA). FIJI ImageJ software [72] was used to evaluate mean gray values (average signal intensity over the selected area) which facilitated quantification of digoxigenin-labeled transcript signals in the brains of Rbfox1.457- or control-treated mosquitoes as described [73]. These transcript data were evaluated with the Student’s *t*-test. 

### 3.7. Evaluation of Non-target Species

Yeast toxicity was assessed as previously described [8,9] in *D. melanogaster, T. castaneum,* and *D. magna. O. fasciatus* and *H. convergens* were evaluated as follows:

#### 3.7.1. *O. fasciatus*

Adults were obtained from Carolina Biologicals (Burlington, NC, USA) and reared according to the provider’s instructions. For the toxicity assays, in each of two biological replicate experiments, 20 individuals were fed a slurry of 200 μL of 10% sucrose mixed with red food dye and 50 mg of either control or Rbfox1.457 yeast. This slurry was provided to the insects via a wick-containing 0.5 mL tube that was suspended inside the cage throughout the trial period. The cages were stored at room temperature (21 °C) and monitored for six days. Feeding was confirmed by observation of individual feeding bouts and the presence of red dye in the insect excrement. The number of adults that survived after six days was recorded, and data were analyzed with the Fisher’s exact text.

#### 3.7.2. *H. convergens*

Adults were obtained from Carolina Biologicals and reared in cages stored at room temperature (21 °C) according to the provider’s instructions. Toxicity assays were conducted as described for *O. fasciatus,* except that the yeast ATSB was provided to 10 insects in a small dish throughout each of two trials. 

## 4. Conclusions

The results of this study suggest that Rbfox1.457 yeast, a dual-action adulticidal and larvicidal IRP with a target site conserved in the *Rbfox1* gene of many species of mosquitoes (Table S1), could potentially be used as a new means of controlling *Aedes, Anopheles,* and *Culex* mosquitoes at multiple stages of the mosquito life cycle (Figure 1, Figure 2 and Figure 4). Silencing *Rbfox1* resulted in significant decreases in *Notch* and *spdo* transcripts, a result that had not previously been described in other species (Figure 3 and Figure S1). This critical role in *Notch* signaling, a key cellular signaling mechanism, correlated with a critical requirement for *Rbfox1* for mosquito viability. These studies also demonstrated that Rbfox1.457 IRPs, which did not impact survival of several non-target arthropods evaluated in this investigation (Table 3), can be effectively delivered to adult mosquitoes in the form of an ATSB (Figure 2 and Figure 4). Discovery that yeast-based IRPs can be delivered to mosquitoes as ATSBs greatly improves the potential for affordable and scalable use of RNAi-based adulticides. Moreover, development and characterization of Rbfox1.457 yeast IRP (Figure 1 and Figure 4) added one more larvicide to the growing collection of larvicidal IRPs, an arsenal which could be used to combat resistance, should it develop, to any single yeast IRP [4,31]. Confirmation of Rbfox1.457 yeast ATSB activity in simulated field trials (Figure 2 and Figure 4) and Rbfox1.457 yeast larvicide activity in semi-field trials (Figure 1d) suggests that IRP technology could potentially be implemented successfully in the field. This will of course need to be evaluated in larger-scale field trials, which should focus on residual activity of the IRP following deployment, a critical factor that must be evaluated in order to completely assess the potential for using these insecticides as a new mosquito control intervention.

Hunter et al. [74] previously demonstrated in a large-scale field trial, under natural beekeeping conditions, that RNAi could be used to prevent *Apis mellifera* infections with Israeli Acute Paralysis Virus. Their study, which was performed in two discrete climates, seasons, and geographical locations, demonstrated that the dsRNA could be successfully delivered in sugar water. However, the bees were treated twice per week during the trial period. Less frequent applications of ATSBs would be ideal for mosquito control, suggesting that the development of long-lasting formulations would be a worthwhile endeavor. The development of encapsulated stable formulations that promote yeast IRP stability in various environmental conditions, both prior to and during use, will be important. Encapsulation of the yeast could also facilitate controlled and extended IRP release, which is likely an essential component of developing commercial products with suitable residual activity [31]. In preparation for large-scale field trials, it will also be useful to scale production of yeast strains that express insecticidal shRNA, and this may require further research and development [31]. The results of this investigation indicate that pursuit of these endeavors could significantly advance mosquito control efforts.

## Figures and Tables

**Table 1:** *Aedes aegypti* mortality induced by Rbfox1.457 siRNA.

Experiment ^1^	% Mortality	*p* Value Control vs. Treatment	n
Larval soaking			
Control siRNA	0 ± 0	4.4^−13^	40
Rbfox1 siRNA	73 ± 2.5		
Adult microinjection			
Control siRNA	5	4.0^−3^	20
Rbfox1 siRNA	45		
ATSB/siRNA feeding			
Control siRNA	8 ± 5.3	3.5^−10^	37
Rbfox1 siRNA	77 ± 7.1		42

^1^ Mortality rates (with standard deviations (SDs) for the soaking and standard errors of the mean (SEMs) for feedings), the total numbers of individuals subjected to each treatment (n), and the *p* value obtained in Fisher’s exact test analyses between Rbfox1.457 siRNA-treated and corresponding control siRNA-treated individuals are indicated.

**Table 2:** ATSB feeding rates in mosquitoes.

		Feeding Rate (%)		
Experiment ^1^	Species	Control	Rbfox1.457	n
siRNA ATSB	*A. aegypti*	57 ± 7	63 ± 8.5	65
Yeast ATSB	*A. aegypti*	100 ± 0	100 ± 0	150
	*A. gambiae*	100 ± 0	100 ± 0	75
	*C. quinquefasciatus*	100 ± 0	100 ± 0	225
	*A. albopictus*	89 ± 0.5	91 ± 1	225

^1^ The percentages of insects that became engorged with sugar meals consisting of each of the indicated treatments are shown. The mosquito species, feeding rates with SEMs, and the total number of individuals subjected to each treatment (n) are indicated. No significant differences between control or insecticidal treatments were observed.

**Table 3:** Survival of select non-target organisms treated with Rbfox1.457.

Test Organism ^1^	Control	Rbfox1.457	n
*D. magna* adults	100 ± 0	98 ± 3.5	40
*D. melanogaster* larvae	100 ± 0	100 ± 0	60
*D. melanogaster* adults	100 ± 0	99 ± 1	60
*H. convergens* adults	90 ± 0	90 ± 7	20
*O. fasciatus* adults	80 ± 7	77.5 ± 18	40
*T. castaneum* adults	100 ± 0	100 ± 0	60

^1^ The percentages of insects that became engorged with sugar meals consisting of each of the indicated treatments (Control or Rbfox1.457). The mosquito species, feeding rates with SDs, and the total number of individuals subjected to each treatment (n) are indicated. No significant differences between control or insecticidal treatments were observed.

## Data Availability

All data is provided within the text and Supplementary Materials.

## Supplementary Materials

The following are available online at https://www.mdpi.com/2076-0817/10/10/1251/s1, Table S1: Conservation of the Rbfox1.457 yeast IRP target site in mosquitoes. The Rbfox1.457 yeast target site is conserved in the *Rbfox1* genes of multiple disease vector mosquitoes. Outside of *H. illucens*, *L. longipalpis*, and *P. papatasi*, no other identical 21 bp target site was found in the genomes of non-target species found in the National Center Biotechnology Information (NCBI) database [27]. Figure S1: Rbfox1.457 yeast ATSB results in target gene silencing and significantly impacts *Notch* signaling in *A. gambiae*. Rbfox1.457 ATSB consumption resulted in a significant reduction in *Rbfox1* (A), *Notch* (B), and *spdo* (C) transcripts in the *A. gambiae* adult female brain. The numbers of brains analyzed (n) are indicated below each bar in the graphs; *** = *p* < 0.001 vs. sugar bait alone or sugar bait with control yeast; data were analyzed with Student’s *t*-test. Figure S2: Original gel for panel 1a (both unlabeled and labeled).

## References

[1] Airs P.M., Bartholomay L.C. (2017). RNA interference for mosquito and mosquito-borne disease control. Insects.

[2] Environmental Protection Agency Pesticides. https://www.epa.gov/pesticides.

[3] World Health Organization (2009). Dengue Guidelines for Diagnosis, Treatment, Prevention and Control.

[4] Wiltshire R.M., Duman-Scheel M. (2020). Advances in oral RNAi for disease vector mosquito research and control. Curr. Opin. Insect Sci..

[5] Zhang J., Khan S.A., Heckel D.G., Bock R. (2017). Next-generation insect-resistant plants: RNAi-mediated crop protection. Trends Biotechnol..

[6] Hapairai L.K., Mysore K., Chen Y., Harper E.I., Scheel M.P., Lesnik A.M., Sun L., Severson D.W., Wei N., Duman-Scheel M. (2017). Lure-and-kill yeast interfering RNA larvicides targeting neural genes in the human disease vector mosquito *Aedes aegypti*. Sci. Rep..

[7] Mysore K., Hapairai L.K., Sun L., Harper E.I., Chen Y., Eggleson K.K., Realey J.S., Scheel N.D., Severson D.W., Wei N. (2017). Yeast interfering RNA larvicides targeting neural genes induce high rates of *Anopheles* larval mortality. Malar. J..

[8] Hapairai L.K., Mysore K., Sun L., Li P., Wang C.W., Scheel N.D., Lesnik A., Scheel M.P., Igiede J., Wei N. (2020). Characterization of an adulticidal and larvicidal interfering RNA pesticide that targets a conserved sequence in mosquito G protein-coupled dopamine 1 receptor genes. Insect Biochem. Mol. Biol..

[9] Mysore K., Hapairai L.K., Sun L., Li P., Wang C.W., Scheel N.D., Lesnik A., Igiede J., Scheel M.P., Wei N. (2020). Characterization of a dual-action adulticidal and larvicidal interfering RNA pesticide targeting the Shaker gene of multiple disease vector mosquitoes. PLoS Negl. Trop. Dis..

[10] Mysore K., Li P., Wang C.W., Hapairai L.K., Scheel N.D., Realey J.S., Sun L., Roethele J.B., Severson D.W., Wei N. (2019). Characterization of a yeast interfering RNA larvicide with a target site conserved in the synaptotagmin gene of multiple disease vector mosquitoes. PLoS Negl. Trop. Dis..

[11] Mysore K., Li P., Wang C.W., Hapairai L.K., Scheel N.D., Realey J.S., Sun L., Severson D.W., Wei N., Duman-Scheel M. (2019). Characterization of a broad-based mosquito yeast interfering RNA larvicide with a conserved target site in mosquito semaphorin-1a genes. Parasit. Vectors.

[12] Conboy J.G. (2017). Developmental regulation of RNA processing by Rbfox proteins. Wiley Interdiscip. Rev. RNA.

[13] Shukla J.P., Deshpande G., Shashidhara L.S. (2017). Ataxin 2-binding protein 1 is a context-specific positive regulator of Notch signaling during neurogenesis in *Drosophila melanogaster*. Development.

[14] Nikonova E., Kao S.Y., Ravichandran K., Wittner A., Spletter M.L. (2019). Conserved functions of RNA-binding proteins in muscle. Int. J. Biochem. Cell Biol..

[15] Shibata H., Huynh D.P., Pulst S.M. (2000). A novel protein with RNA-binding motifs interacts with ataxin-2. Hum. Mol. Genet..

[16] Gazzara M.R., Mallory M.J., Roytenberg R., Lindberg J.P., Jha A., Lynch K.W., Barash Y. (2017). Ancient antagonism between CELF and RBFOX families tunes mRNA splicing outcomes. Genome Res..

[17] Kucherenko M.M., Shcherbata H.R. (2018). Stress-dependent miR-980 regulation of Rbfox1/A2bp1 promotes ribonucleoprotein granule formation and cell survival. Nat. Commun..

[18] Tastan O.Y., Maines J.Z., Li Y., McKearin D.M., Buszczak M. (2010). *Drosophila* ataxin 2-binding protein 1 marks an intermediate step in the molecular differentiation of female germline cysts. Development.

[19] Carreira-Rosario A., Bhargava V., Hillebrand J., Kollipara R.K., Ramaswami M., Buszczak M. (2016). Repression of Pumilio protein expression by Rbfox1 promotes germ cell differentiation. Dev. Cell.

[20] Bajpai R., Sambrani N., Stadelmayer B., Shashidhara L.S. (2004). Identification of a novel target of D/V signaling in *Drosophila* wing disc: Wg-independent function of the organizer. Gene Expr. Patterns.

[21] Usha N., Shashidhara L.S. (2010). Interaction between Ataxin-2 Binding Protein 1 and Cubitus-interruptus during wing development in *Drosophila*. Dev. Biol..

[22] Guven-Ozkan T., Busto G.U., Schutte S.S., Cervantes-Sandoval I., O’Dowd D.K., Davis R.L. (2016). MiR-980 is a memory suppressor microRNA that regulates the autism-susceptibility gene A2bp1. Cell Rep..

[23] Nazario-Toole A.E., Robalino J., Okrah K., Corrada-Bravo H., Mount S.M., Wu L.P. (2018). The splicing factor RNA-binding Fox Protein 1 mediates the cellular immune response in *Drosophila melanogaster*. J. Immunol..

[24] Koizumi K., Higashida H., Yoo S., Islam M.S., Ivanov A.I., Guo V., Pozzi P., Yu S.H., Rovescalli A.C., Tang D. (2007). RNA interference screen to identify genes required for *Drosophila* embryonic nervous system development. Proc. Natl. Acad. Sci. USA.

[25] Tunstall N.E., Herr A., de Bruyne M., Warr C.G. (2012). A screen for genes expressed in the olfactory organs of *Drosophila melanogaster* identifies genes involved in olfactory behaviour. PLoS ONE.

[26] Giraldo-Calderon G.I., Emrich S.J., MacCallum R.M., Maslen G., Dialynas E., Topalis P., Ho N., Gesing S., VectorBase C., Madey G. (2015). VectorBase: An updated bioinformatics resource for invertebrate vectors and other organisms related with human diseases. Nucleic Acids Res..

[27] Sayers E.W., Beck J., Bolton E.E., Bourexis D., Brister J.R., Canese K., Comeau D.C., Funk K., Kim S., Klimke W. (2021). Database resources of the National Center for Biotechnology Information. Nucleic Acids Res..

[28] Sousa-Paula L.C., Otranto D., Dantas-Torres F. (2020). *Lutzomyia longipalpis* (Sand Fly). Trends Parasitol..

[29] Fiorenzano J.M., Koehler P.G., Xue R.D. (2017). Attractive toxic sugar bait (ATSB) for control of mosquitoes and its impact on non-target organisms: A review. Int. J. Environ. Res. Public Health.

[30] Faraji A., Unlu I. (2016). The eye of the tiger, the thrill of the gight: Effective larval and adult control measures against the Asian tiger mosquito, *Aedes albopictus* (Diptera: Culicidae), in North America. J. Med. Entomol..

[31] Duman-Scheel M. (2019). *Saccharomyces cerevisiae* (baker’s yeast) as an interfering RNA expression and delivery system. Curr. Drug Targets.

[32] Mysore K., Hapairai L.K., Li P., Roethele J.B., Sun L., Igiede J., Misenti J.K., Duman-Scheel M. (2021). A functional requirement for sex-determination M/m locus region lncRNA genes in *Aedes aegypti* female larvae. Sci. Rep..

[33] Mysore K., Sun L., Roethele J.B., Li P., Igiede J., Misenti J.K., Duman-Scheel M. (2021). A conserved female-specific larval requirement for MtnB function facilitates sex separation in multiple species of disease vector mosquitoes. Parasit. Vectors.

[34] Skeath J.B., Doe C.Q. (1998). Sanpodo and Notch act in opposition to Numb to distinguish sibling neuron fates in the *Drosophila* CNS. Development.

[35] Mulla M.S. (1967). Larvicides and larvicidal formulations for the control of *Culex pipiens fatigans*. Bull. World Health Organ..

[36] World Health Organization (2013). Larval Source Management: A Supplementary Measure for Malaria Vector Control: An Operational Manual.

[37] Center for Disease Control Larvicides. https://www.cdc.gov/mosquitoes/mosquito-control/community/larvicides.html.

[38] Afrane Y.A., Mweresa N.G., Wanjala C.L., Gilbreath III T.M., Zhou G., Lee M.C., Githeko A.K., Yan G. (2016). Evaluation of long-lasting microbial larvicide for malaria vector control in Kenya. Malar. J..

[39] Kahindi S.C., Muriu S., Derua Y.A., Wang X., Zhou G., Lee M.C., Mwangangi J., Atieli H., Githeko A.K., Yan G. (2018). Efficacy and persistence of long-lasting microbial larvicides against malaria vectors in western Kenya highlands. Parasit. Vectors.

[40] Derua Y.A., Kweka E.J., Kisinza W.N., Githeko A.K., Mosha F.W. (2019). Bacterial larvicides used for malaria vector control in sub-Saharan Africa: Review of their effectiveness and operational feasibility. Parasit. Vectors.

[41] Derua Y.A., Kahindi S.C., Mosha F.W., Kweka E.J., Atieli H.E., Zhou G., Lee M.C., Githeko A.K., Yan G. (2019). Susceptibility of *Anopheles gambiae* complex mosquitoes to microbial larvicides in diverse ecological settings in western Kenya. Med. Vet. Entomol..

[42] Getachew D., Balkew M., Tekie H. (2020). *Anopheles* larval species composition and characterization of breeding habitats in two localities in the Ghibe River Basin, southwestern Ethiopia. Malar. J..

[43] Khallaayoune K., Qualls W.A., Revay E.E., Allan S.A., Arheart K.L., Kravchenko V.D., Xue R.D., Schlein Y., Beier J.C., Muller G.C. (2013). Attractive toxic sugar baits: Control of mosquitoes with the low-risk active ingredient dinotefuran and potential impacts on nontarget organisms in Morocco. Environ. Entomol..

[44] Qualls W.A., Muller G.C., Revay E.E., Allan S.A., Arheart K.L., Beier J.C., Smith M.L., Scott J.M., Kravchenko V.D., Hausmann A. (2014). Evaluation of attractive toxic sugar bait (ATSB)-barrier for control of vector and nuisance mosquitoes and its effect on non-target organisms in sub-tropical environments in Florida. Acta Trop..

[45] Fulcher A., Scott J.M., Qualls W.A., Muller G.C., Xue R.D. (2014). Attractive toxic sugar baits mixed with pyriproxyfen sprayed on plants against adult and larval *Aedes albopictus* (Diptera: Culicidae). J. Med. Entomol..

[46] Revay E.E., Muller G.C., Qualls W.A., Kline D.L., Naranjo D.P., Arheart K.L., Kravchenko V.D., Yefremova Z., Hausmann A., Beier J.C. (2014). Control of *Aedes albopictus* with attractive toxic sugar baits (ATSB) and potential impact on non-target organisms in St. Augustine, Florida. Parasitol. Res..

[47] Junnila A., Revay E.E., Muller G.C., Kravchenko V., Qualls W.A., Xue R.D., Allen S.A., Beier J.C., Schlein Y. (2015). Efficacy of attractive toxic sugar baits (ATSB) against *Aedes albopictus* with garlic oil encapsulated in beta-cyclodextrin as the active ingredient. Acta Trop..

[48] Seeger K.E., Scott J.M., Muller G.C., Qualls W.A., Xue R.D. (2017). Effect of common species of Florida landscaping plants on the efficacy of attractive toxic sugar baits against *Aedes albopictus*. J. Am. Mosq. Control. Assoc..

[49] Sippy R., Rivera G.E., Sanchez V., Heras F., Morejon B., Beltran E., Hikida R.S., Lopez-Latorre M.A., Aguirre A., Stewart-Ibarra A.M. (2020). Ingested insecticide to control *Aedes aegypti*: Developing a novel dried attractive toxic sugar bait device for intra-domiciliary control. Parasit. Vectors.

[50] Muller G.C., Junnila A., Schlein Y. (2010). Effective control of adult *Culex pipiens* by spraying an attractive toxic sugar bait solution in the vegetation near larval habitats. J. Med. Entomol..

[51] Muller G.C., Junnila A., Qualls W., Revay E.E., Kline D.L., Allan S., Schlein Y., Xue R.D. (2010). Control of *Culex quinquefasciatus* in a storm drain system in Florida using attractive toxic sugar baits. Med. Vet. Entomol..

[52] Qualls W.A., Scott-Fiorenzano J., Muller G.C., Arheart K.L., Beier J.C., Xue R.D. (2016). Evaluation and adaptation of attractive toxic sugar baits for *Culex tarsalis* and *Culex quinquefasciatus* control in the Coachella Valley, Southern California. J. Am. Mosq. Control. Assoc..

[53] Beier J.C., Muller G.C., Gu W., Arheart K.L., Schlein Y. (2012). Attractive toxic sugar bait (ATSB) methods decimate populations of *Anopheles* malaria vectors in arid environments regardless of the local availability of favoured sugar-source blossoms. Malar. J..

[54] Qualls W.A., Muller G.C., Traore S.F., Traore M.M., Arheart K.L., Doumbia S., Schlein Y., Kravchenko V.D., Xue R.D., Beier J.C. (2015). Indoor use of attractive toxic sugar bait (ATSB) to effectively control malaria vectors in Mali, West Africa. Malar. J..

[55] Tenywa F.C., Kambagha A., Saddler A., Maia M.F. (2017). The development of an ivermectin-based attractive toxic sugar bait (ATSB) to target *Anopheles arabiensis*. Malar. J..

[56] Furnival-Adams J.E.C., Camara S., Rowland M., Koffi A.A., Ahoua Alou L.P., Oumbouke W.A., N’Guessan R. (2020). Indoor use of attractive toxic sugar bait in combination with long-lasting insecticidal net against pyrethroid-resistant *Anopheles gambiae*: An experimental hut trial in Mbe, central Cote d’Ivoire. Malar. J..

[57] Traore M.M., Junnila A., Traore S.F., Doumbia S., Revay E.E., Kravchenko V.D., Schlein Y., Arheart K.L., Gergely P., Xue R.D. (2020). Large-scale field trial of attractive toxic sugar baits (ATSB) for the control of malaria vector mosquitoes in Mali, West Africa. Malar. J..

[58] Diarra R.A., Traore M.M., Junnila A., Traore S.F., Doumbia S., Revay E.E., Kravchenko V.D., Schlein Y., Arheart K.L., Gergely P. (2021). Testing configurations of attractive toxic sugar bait (ATSB) stations in Mali, West Africa, for improving the control of malaria parasite transmission by vector mosquitoes and minimizing their effect on non-target insects. Malar. J..

[59] MonSanto Docket ID: EPA-HQ-OPP-2013-0485. https://www.apsnet.org/members/outreach/ppb/Documents/Monsanto%20posted%20written%20comment%20Jan%202014.pdf.

[60] Casanova C., Andrighetti M.T., Sampaio S.M., Marcoris M.L., Colla-Jacques F.E., Prado A.P. (2013). Larval breeding sites of *Lutzomyia longipalpis* (Diptera: Psychodidae) in visceral leishmaniasis endemic urban areas in Southeastern Brazil. PLoS Negl. Trop. Dis..

[61] Vivero R.J., Torres-Gutierrez C., Bejarano E.E., Pena H.C., Estrada L.G., Florez F., Ortega E., Aparicio Y., Muskus C.E. (2015). Study on natural breeding sites of sand flies (Diptera: Phlebotominae) in areas of *Leishmania* transmission in Colombia. Parasit. Vectors.

[62] Ferreira T.N., Pita-Pereira D., Costa S.G., Brazil R.P., Moraes C.S., Diaz-Albiter H.M., Genta F.A. (2018). Transmission blocking sugar baits for the control of *Leishmania* development inside sand flies using environmentally friendly beta-glycosides and their aglycones. Parasit. Vectors.

[63] McDermott E.G., Morris E.K., Garver L.S. (2019). Sodium ascorbate as a potential toxicant in attractive sugar baits for control of adult mosquitoes (Diptera: Culicidae) and sand flies (Diptera: Psychodidae). J. Med. Entomol..

[64] Clemons A., Mori A., Haugen M., Severson D.W., Duman-Scheel M. (2010). Culturing and egg collection of *Aedes aegypti*. Cold Spring Harb. Protoc..

[65] Singh A.D., Wong S., Ryan C.P., Whyard S. (2013). Oral delivery of double-stranded RNA in larvae of the yellow fever mosquito, *Aedes aegypti: Implications* for pest mosquito control. J. Insect Sci..

[66] World Health Organization (2005). Guidelines for Laboratory and Field Testing of Mosquito Larvicides.

[67] Tomchaney M., Mysore K., Sun L., Li P., Emrich S.J., Severson D.W., Duman-Scheel M. (2014). Examination of the genetic basis for sexual dimorphism in the *Aedes aegypti* (dengue vector mosquito) pupal brain. Biol. Sex. Differ..

[68] van Dijken J.P., Bauer J., Brambilla L., Duboc P., Francois J.M., Gancedo C., Giuseppin M.L., Heijnen J.J., Hoare M., Lange H.C. (2000). An interlaboratory comparison of physiological and genetic properties of four *Saccharomyces cerevisiae* strains. Enzyme Microb. Technol..

[69] Mysore K., Hapairai L.K., Wei N., Realey J.S., Scheel N.D., Severson D.W., Duman-Scheel M. (2019). Preparation and use of a yeast shRNA delivery system for gene silencing in mosquito larvae. Methods Mol. Biol..

[70] Patel N.H., Krieg P.A. (1996). In situ hybridization to whole mount *Drosophila* embryos. A Laboratory Guide to RNA: Isolation, Analysis, and Synthesis.

[71] Haugen M., Tomchaney M., Kast K., Flannery E., Clemons A., Jacowski C., Simanton Holland W., Le C., Severson D., Duman-Scheel M. (2010). Whole-mount in situ hybridization for analysis of gene expression during *Aedes aegypti* development. Cold Spring Harb. Protoc..

[72] Schindelin J., Arganda-Carreras I., Frise E., Kaynig V., Longair M., Pietzsch T., Preibisch S., Rueden C., Saalfeld S., Schmid B. (2019). Fiji: An open-source platform for biological-image analysis. Nat. Methods.

[73] Mysore K., Sun L., Tomchaney M., Sullivan G., Adams H., Piscoya A.S., Severson D.W., Syed Z., Duman-Scheel M. (2015). siRNA-Mediated silencing of doublesex during female development of the dengue vector mosquito *Aedes aegypti*. PLoS Negl. Trop. Dis..

[74] Hunter W., Ellis J., Vanengelsdorp D., Hayes J., Westervelt D., Glick E., Williams M., Sela I., Maori E., Pettis J. (2010). Large-scale field application of RNAi technology reducing Israeli acute paralysis virus disease in honey bees (*Apis mellifera,* Hymenoptera: Apidae). PloS Pathog..

