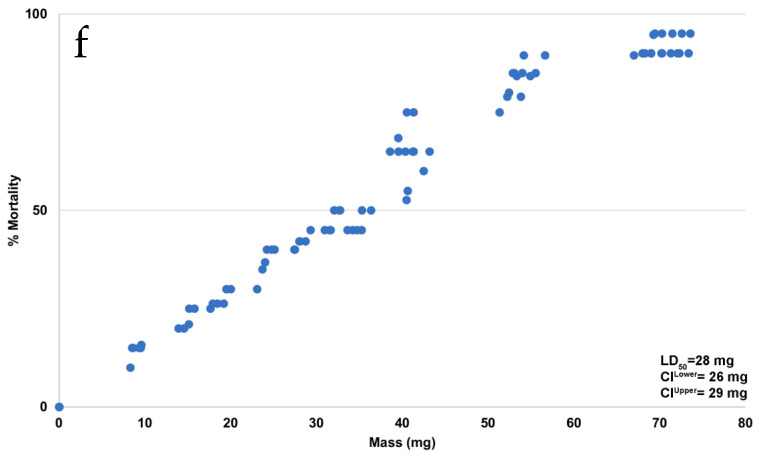# Correction: Mysore et al. A Broad-Based Mosquito Yeast Interfering RNA Pesticide Targeting *Rbfox1* Represses *Notch* Signaling and Kills Both Larvae and Adult Mosquitoes. *Pathogens* 2021, *10*, 1251

**DOI:** 10.3390/pathogens11090956

**Published:** 2022-08-23

**Authors:** Keshava Mysore, Longhua Sun, Limb K. Hapairai, Chien-Wei Wang, Joseph B. Roethele, Jessica Igiede, Max P. Scheel, Nicholas D. Scheel, Ping Li, Na Wei, David W. Severson, Molly Duman-Scheel

**Affiliations:** 1 Department of Medical and Molecular Genetics, Indiana University School of Medicine, Raclin-Carmichael Hall, 1234 Notre Dame Ave., South Bend, IN 46617, USA; 2 Eck Institute for Global Health, The University of Notre Dame, South Bend, IN 46556, USA; 3 Department of Civil and Environmental Engineering and Earth Sciences, The University of Notre Dame, South Bend, IN 46556, USA; 4 Department of Biological Sciences, The University of Notre Dame, South Bend, IN 46556, USA; 5 Department of Life Sciences, The University of the West Indies, St. Augustine, Trinidad and Tobago

**Error in Figure**

In the original publication [1], there was a mistake in Figure 1 as published. The wrong graph was inadvertently included in panel 1f (dose–response curve). Additionally, the original image for the gel shown in panel 1a is now included in the Supplementary Materials. The corrected panel 1f appears below, and the original gel for panel 1a (both unlabeled and labeled) is updated in Supplementary Materials. The authors apologize for any inconvenience caused and state that the scientific conclusions are unaffected. This correction was approved by the Academic Editor. The original publication has also been updated.