# Supplementary material for: A Broad-Based Mosquito Yeast Interfering RNA Pesticide Targeting Rbfox1 Represses Notch Signaling and Kills Both Larvae and Adult Mosquitoes
Source: Pathogens. 2021 Sep 28;10(10):1251. doi: 10.3390/pathogens10101251 (PMC8541554; doi:10.3390/pathogens10101251)
Supplement: Supplementary file 1 [file pathogens-10-01251-s001.zip › Table S1.pdf]

Table S1. Conservation of the Rbfox1.457 target site in mosquitoes.

Species or Taxon Group	Gene or Contig
Aedes aegypti	AAEL019934
Aedes albopictus	LOC109398542/LOC109419707
Anopheles arabiensis	AARA017873
Anopheles christyi	KB698817
Anopheles coluzzii	ACOM024690/ACON006089
Anopheles culicafacies	KI422895
Anopheles dirus	ADIR008834
Anopheles epiroticus	AEPI000570
Anopheles funestus	AFUN020876
Anopheles maculatus	KI437899
Anopheles gambiae	AGAP006089
Anopheles melas	KI920132
Anopheles merus	LOC121593792
Anopheles minimus	AMIN009931
Anopheles quadriannulatus	AQUA017390
Anopheles sinensis	KI916227
Anopheles stephensi	LOC118510461
Culex pipiens pallens	LOC120425920
Culex quinquefasciatus	LOC6044582
Hermetia illucens	LOC119656183/LOC119647768
Lutzomyia longipalpis	LLOJ006090
Phlebotomus papatasi	JP545859.1
Amphibians	None
Birds	None
Fish	None
Fungi	None
Human	None
Mammals	None
Plants	None
Reptiles	None
