# Supplementary figures and images for: A Broad-Based Mosquito Yeast Interfering RNA Pesticide Targeting Rbfox1 Represses Notch Signaling and Kills Both Larvae and Adult Mosquitoes
Source: Pathogens. 2021 Sep 28;10(10):1251. doi: 10.3390/pathogens10101251 (PMC8541554; doi:10.3390/pathogens10101251)

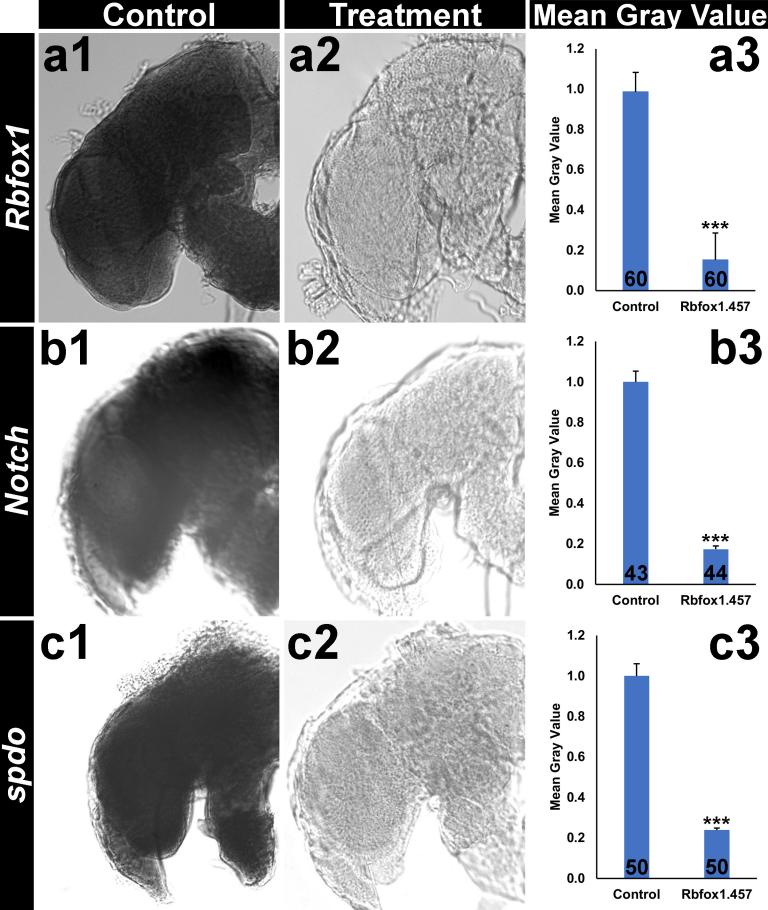

Supplement: Supplementary file 1 [file pathogens-10-01251-s001.zip › Figure S1.pdf]
